# Usefulness of the Hybrid RFR-FFR Approach: Results of a Prospective and Multicenter Analysis of Diagnostic Agreement between RFR and FFR—The RECOPA (REsting Full-Cycle Ratio Comparation versus Fractional Flow Reserve (A Prospective Validation)) Study

**DOI:** 10.1155/2021/5522707

**Published:** 2021-03-31

**Authors:** Juan Casanova-Sandoval, Diego Fernández-Rodríguez, Imanol Otaegui, Teresa Gil Jiménez, Marcos Rodríguez-Esteban, Kristian Rivera, Francisco Torres-Saura, Víctor Jiménez Díaz, Raymundo Ocaranza-Sánchez, Vicente Peral Disdier, Guillermo Sánchez-Elvira, Fernando Worner

**Affiliations:** ^1^Hospital Universitari Arnau de Vilanova, Lleida, Spain; ^2^Institut de Recerca Biomédica de Lleida (IRBLleida), Lleida, Spain; ^3^Hospital Universitari Vall d´Hebron, Barcelona, Spain; ^4^Hospital Universitario Clínico San Cecilio de Granada, Granada, Spain; ^5^Hospital Universitario Nuestra Señora de Candelaria, Tenerife, Spain; ^6^Hospital Universitario de Vinalopó, Elche, Spain; ^7^Hospital Universitario Alvaro Cunqueiro, Vigo, Spain; ^8^Hospital Universitario Lucus Augusti, Lugo, Spain; ^9^Hospital Son Espases, Palma de Mallorca, Spain; ^10^Complejo Hospitalario de Navarra, Pamplona, Spain

## Abstract

**Background:**

The resting full‐cycle ratio (RFR) is a novel resting index which in contrast to the gold standard (fractional flow reserve (FFR)) does not require maximum hyperemia induction. The objectives of this study were to evaluate the agreement between RFR and FFR with the currently recommended thresholds and to design a hybrid RFR-FFR ischemia detection strategy, allowing a reduction of coronary vasodilator use.

**Materials and Methods:**

Patients subjected to invasive physiological study in 9 Spanish centers were prospectively recruited between April 2019 and March 2020. Sensitivity and specificity studies were made to assess diagnostic accuracy between the recommended levels of RFR ≤0.89 and FFR ≤0.80 (primary objective) and to determine the RFR “grey zone” in order to define a hybrid strategy with FFR affording 95% global agreement compared with FFR alone (secondary objective).

**Results:**

A total of 380 lesions were evaluated in 311 patients. Significant correlation was observed (*R*^2^ = 0.81; *P* < 0.001) between the two techniques, with 79% agreement between RFR ≤ 0.89 and FFR ≤ 0.80 (positive predictive value, 68%, and negative predictive value, 80%). The hybrid RFR-FFR strategy, administering only adenosine in the “grey zone” (RFR: 0.86 to 0.92), exhibited an agreement of over 95% with FFR, with high predictive values (positive predictive value, 91%, and negative predictive value, 92%), reducing the need for vasodilators by 58%.

**Conclusions:**

Dichotomous agreement between RFR and FFR with the recommended thresholds is significant but limited. The adoption of a hybrid RFR-FFR strategy affords very high agreement, with minimization of vasodilator use.

## 1. Introduction

Fractional flow reserve (FFR) is the coronary resistance index with the greatest body of supporting evidence and is considered the gold standard in the invasive detection of ischemia [1–5]. Nonhyperemic pressure ratios (NHPRs) that do not require maximum hyperemia induction have gradually been introduced, with the instantaneous wave-free ratio (iFR) being the most widely used index [6, 7].

The resting full-cycle ratio (RFR) is a new NHPR that assesses the hemodynamic significance of coronary stenoses, identifying the lowest distal arterial pressure (Pd)/arterial pressure (Pa) ratio over the entire cardiac cycle. In contrast to other NHPRs, its measurements would be independent of the morphology of the pressure waves, the electrical signal, and the phasic variations in microcirculatory resistance [8, 9]. Initial validation of the RFR was performed by Svanerud et al. [8], showing RFR values ≤0.89 to be in good agreement with iFR values ≤0.89, through indirect analysis of registries from other studies [10–13]. Recently, Kumar et al. have again validated these RFR thresholds against iFR [14]. However, the validation of diagnostic tests without comparison against the gold standard, using data from nonspecifically designed studies and choosing dichotomous thresholds, may limit assessment of the usefulness of a diagnostic test.

Therefore, a specifically designed, prospective multicenter study [the RECOPA (*RE*sting full-cycle ratio *CO*mparation versus fractional flow reserve: a *P*rospective v*A*lidation) Study] was carried out to directly assess global agreement of the recommended values of RFR (≤0.89) and FFR (≤0.80). We likewise compared the usefulness of a hybrid RFR and FFR guided ischemia detection strategy versus a strategy guided by FFR alone in reducing the need for coronary vasodilators, maintaining high agreement.

## 2. Materials and Methods

### 2.1. Study Population

In the period between April 2019 and March 2020, in 9 Spanish centers, we prospectively recruited patients with ischemic heart disease referred to the hemodynamics laboratory for diagnostic coronary angiography, in which functional assessment of the coronary lesions was considered necessary. Patients with both intermediate coronary lesions (40%–69%) and severe coronary lesions (≥70%) were included, and invasive pressure-guided physiological coronary studies were performed to assess RFR and FFR values. If ad hoc percutaneous coronary intervention was proved necessary, the clinical decision was made based on the result corresponding to FFR.

The eligibility criteria are detailed below.

#### 2.1.1. Inclusion Criteria

(1) Age ≥ 18 years
(2) Coronary lesions amenable to invasive physiological evaluation in patients
    (i) With stable ischemic heart disease
    (ii) With culprit lesions in non-ST segment elevation acute coronary syndrome (NSTEACS)
    (iii) With nonculprit lesions in NSTEACS
    (iv) With nonculprit lesions in ST segment elevation acute coronary syndrome (STEACS) subjected to second step evaluation

#### 2.1.2. Exclusion Criteria

1. Allergy to the contrast medium not amenable to premedication
2. Severe bronchial asthma or intolerance to adenosine
3. Atrioventricular block (≥ second grade)
4. Cardiogenic shock
5. Women of child-bearing potential
6. Any other medical condition which in the opinion of the investigator could pose patient safety problems or alter the study results

### 2.2. Diagnostic Procedure

Following diagnostic coronary angiography, an analysis of coronary lesions, in terms of percentage of stenosis and length of the lesion by visual estimation, was performed. Subsequently, a pressure-guided functional assessment of the coronary lesions was carried out, first measuring RFR and then FFR, in order to avoid the interference of coronary vasodilatation on RFR values. More than one lesion could be evaluated in the same patient. The measurements were obtained positioning the PressureWire™ X Guidewire 0.014 (Abbott Vascular Inc., Santa Clara, CA, USA) distal to the lesion. Adenosine, the most widely used vasodilator, could be administered via both the intravenous and intracoronary routes. The recommendations for conduction of invasive studies are specified more in detail in Supplementary Material 1.

### 2.3. Study Variables and Objectives

The RFR values considered to be positive for ischemia were ≤0.89. With regard to the gold standard, the FFR values considered to be positive for ischemia were ≤0.80. Based on these values, the primary study objective was to dichotomously determine the diagnostic accuracy of RFR ≤ 0.89 against FFR ≤ 0.80.

Due to the inherent variability of the sensitivity and specificity values of the selected cut-off points, the secondary study objective was to determine an interval of RFR values affording high agreement, making it possible to reduce the administration of vasodilators in the context of a hybrid RFR-FFR ischemia detection strategy versus a strategy guided by FFR alone.

### 2.4. Data Collection and Ethical Considerations

All data were compiled on a prospective basis and entered into a specifically designed database. Each center entered demographic, clinical, laboratory test, angiographic, and physiological data in the database. The study was approved by the Clinical Research Ethics Committee of each center and abided with the requirements and standards of the Declaration of Helsinki and its subsequent amendments regarding research studies in humans, as well as with the data protection regulations applicable in Spain.

### 2.5. Statistical Analysis

The statistical analyses were performed using the R version 3.4.2 package (R Foundation for Statistical Computing, Vienna, Austria), with statistical significance being considered for *p* < 0.05. Categorical variables were reported as numbers and relative frequencies (percentages) and continuous variables as the mean (standard deviation [SD]) or median and range or interquartile range (IQR), depending on their distribution, which was assessed using the Kolmogorov–Smirnov test. The data were evaluated per patient for clinical variables and per lesion for the angiographic and physiological characteristics.

Sensitivity, specificity, and diagnostic accuracy analyses were made in relation to the primary study objective, that is, evaluation of the agreement of the recommended values of RFR (≤0.89) and FFR (≤0.80). Likewise, we estimated the optimum cut-off point of RFR in our sample for an FFR value (≤0.80), based on analysis of the receiver operating characteristic (ROC) curves, with determination of the Youden index. The coefficient of determination (*R*^2^) was also estimated.

In relation to the secondary study objective, exploratory analyses were made to define an interval of values capable of optimizing the positive and negative predictive values (PPV and NPV) of RFR, evaluating the global agreement between a hybrid RFR-FFR strategy and an exclusive FFR strategy in determining the functionally significant lesions. A total agreement of at least 95% was considered ideal.

## 3. Results

A total of 311 patients and 380 lesions were included in the RECOPA Study. A single lesion was examined in most patients, with 5 being the maximum number of lesions evaluated in one patient.

### 3.1. Baseline Clinical Characteristics

The baseline clinical characteristics are described in Table 1. The mean patient age was 65.4 ± 11.5 years; 19.6% of the patients were women and 35.7% were diabetic. On the other hand, 66.2% of the measurements were made in the clinical setting of stable ischemic heart disease and 33.8% in the setting of acute coronary syndrome.

### 3.2. Angiographic Characteristics of the Coronary Lesions

The angiographic characteristics of the coronary lesions are described in Table 2 and Supplementary Material 2. The mean angiographic stenosis was 58 ± 11%, with a reference vessel diameter of 3.02 ± 0.53 mm. The main artery evaluated was the left anterior descending artery, with 59.2% of the measurements, followed by the right coronary artery with 21.6%, the left circumflex artery with 18.2%, and the left main coronary artery with 1.1%. Most of the lesions were under 12 mm in length.

### 3.3. Physiological Characteristics of the Coronary Lesions

The physiological characteristics of the coronary lesions and final treatment are described in Table 3. Most procedures were performed with a 6 F guide catheter (96.8%), using adenosine via the intracoronary (i.c.) route (67.1%). The median RFR was 0.91 (range: 0.86–0.95), the median Pd/Pa at baseline was 0.93 (0.90–0.96), and the median FFR after adenosine administration was 0.84 (0.77–0.89). For the recommended cut-off value of RFR (≤0.89), the total proportion of positive values was 40.0%, versus 35.8% for FFR (cut-off value ≤ 0.80).

### 3.4. Agreement between the Recommended Values of RFR (≤0.89) and FFR (≤0.80)

**Figure 1:**
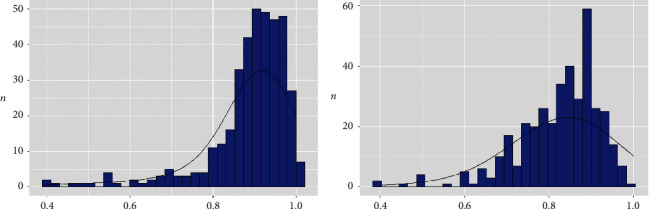
Histograms of the distribution of the RFR and FFR values. (a) RFR; (b) FFR.

**Figure 2:**
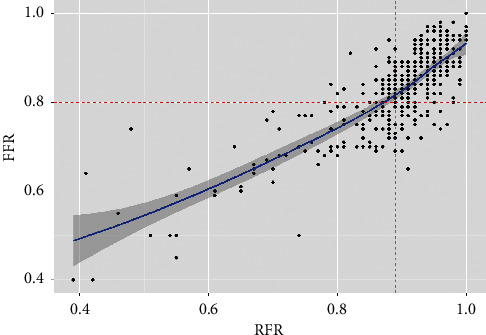
Distribution of the lesions according the RFR and FFR for the recommended cut-off points (RFR ≤ 0.89 and FFR ≤ 0.80). The RFR and FFR values showed a significant correlation (*R*^2^ = 0.81; *P* < 0.001). For the recommended cut-off points of the RFR (≤0.89) and FFR (≤0.80), the following values were obtained: diagnostic accuracy: 0.79; sensitivity: 0.76; specificity: 0.80; positive predictive value: 0.68; negative predictive value: 0.86.

Figure 1 shows the histograms of the distribution of the RFR and FFR values.  Figure 2 in turn shows the distribution of the RFR and FFR values after the administration of adenosine for each lesion; a significant correlation was observed between the two measures (*R*^2^ = 0.81; *P* < 0.001). For the recommended values (RFR ≤ 0.89 and FFR ≤ 0.80), the diagnostic accuracy was 79%. The sensitivity and specificity values were 76% and 80%, respectively, with a PPV of 0.68 and an NPV of 0.86.

The overall analysis for sensitivity and specificity and the stratified analyses according to the route of adenosine administration are shown in Supplementary Material 3.

### 3.5. Optimal Cut-Off Point of RFR

**Figure 3:**
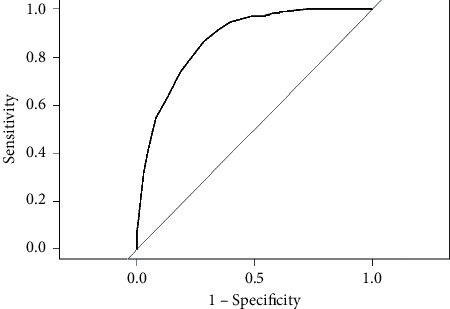
ROC curve of RFR versus FFR ≤0.80. The ROC curve showed an AUC of 0.873 (0.836–0.911; *P* < 0.001). The optimal cut-off point was RFR ≤0.88, showing a Youden index of 0.59 and the following values: diagnostic accuracy: 0.81, sensitivity: 0.71; specificity: 0.87; positive predictive value: 0.75; negative predictive value: 0.84.

The ROC curve (Figure 3) presented an area under the curve (AUC) of 0.873 (0.836–0.911; *P* < 0.001). We found RFR ≤ 0.88 to have the greatest discriminant power in determining FFR ≤0.80, with a Youden index of 0.59. The mentioned value had a diagnostic accuracy of 81%, with a sensitivity of 71% and a specificity of 87%. The PPV was 0.75 and the NPV was 0.84.

### 3.6. Comparison between the Hybrid RFR-FFR Strategy versus the Exclusive FFR Strategy

**Figure 4:**
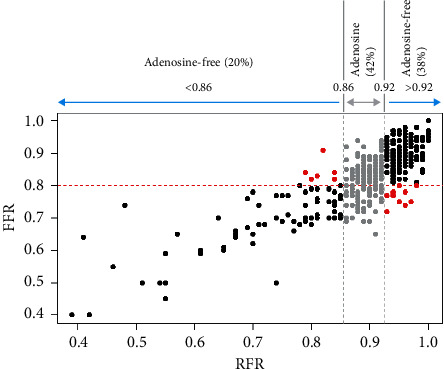
Comparison of the hybrid RFR-FFR strategy versus the exclusive FFR strategy for an agreement of at least 95%. The hybrid RFR-FFR strategy only misclassified 18 lesions (7 false positives and 11 false negatives), diminishing the percentage of lesions requiring the administration of vasodilators to 42% (158 lesions from a total of 380 lesions) compared to the exclusive FFR strategy. Red dots represent the disagreement and black dots represent the agreement between the two strategies. Grey dots should be reclassified by administration of vasodilators and determination of FFR. Two adenosine-free zones (blue) were established (RFR < 0.86 and RFR < 0.92). The adenosine zone (grey) falls between the RFR values of 0.86 and 0.92, with both included.

Figure 4 shows the dispersion plot related to hybrid RFR-FFR strategy compared to FFR-only strategy. Two adenosine-free zones (blue) were established, one below 0.86 and the other above 0.92. The adenosine zone (grey) lies between RFR values of 0.86 and 0.92, with both included. A PPV of 0.91 was obtained for the lower limit (RFR < 0.86), with an NPV of 0.92 for the upper limit (RFR > 0.92). This yielded a global agreement of 95.3% between the two strategies (with only 18 lesions being erroneously classified out of a total of 380 lesions: 7 false positive and 11 false negative). Compared to a FFR-only strategy that would require vasodilator administration in all lesions, the hybrid RFR-FFR strategy would require vasodilator administration in the “adenosine zone,” representing only 42% of the measurements (158 lesions). 58% of the lesions (222 lesions from a total of 380 lesions) in the adenosine-free zones would not require vasodilators use (77 lesions [20% of the total] below RFR < 0.86 and 145 lesions [38% of the total] above RFR > 0.92).

## 4. Discussion

The main findings of the present study were the following: (a) agreement between the recommended dichotomous values for RFR (≤0.89) and FFR (≤0.80) is limited; and (b) a hybrid RFR-FFR strategy for ischemia detection would result in very high agreement compared with an FFR guided strategy alone.

### 4.1. Design and External Validity of the RECOPA Study

The studies mainly conducted by Pijls and De Bruyne on coronary indices served to establish the physiological bases of FFR, determine the expected cut-off points for the FFR technique, and allow its subsequent prospective validation, integrating information from different ischemia detection tests through a prospective multitesting Bayesian approach [1–3]. Thus, the authors were able to establish FFR as the gold standard for the detection of myocardial ischemia related to coronary stenosis [3].

Research on NHPRs has grown markedly in recent years, particularly in relation to iFR, with evidence of the noninferiority of a revascularization strategy guided by iFR versus FFR in two randomized clinical trials [6, 7]. However, the lack of a validation strategy similar to FFR in the different resting indices continues to generate controversy as to whether NHPRs are able to replace FFR as reference method for the invasive detection of myocardial ischemia and thus for the validation of new coronary indices.

Initial RFR validation was made retrospectively and indirectly with respect to iFR [8]. The RFR threshold obtained (≤0.89) was subsequently warranted by Kumar et al. on a prospective basis likewise against iFR [14]. However, the validation of one NHPR against another instead of against the gold standard (FFR) may limit validation. Although Lee et al. [9] reported excellent agreement among a number of resting indices (RFR, iFR, and diastolic pressure ratio) and between these indices and FFR, the data were obtained indirectly from information from other studies [15–18]. To date, only two small studies have evaluated the degree of agreement between RFR and FFR [19, 20]. For this reason, the RECOPA prospective, multicenter validation study was specifically designed to assess agreement between the recommended cut-off values for RFR and FFR in different clinical scenarios with a large sample of patients and thus could provide valuable information.

We consider the external validity of our results to be high. This is because the inclusion criteria used were scantly restrictive, allowing the inclusion of patients with ischemic heart disease encompassing the entire spectrum of situations in which invasive physiological studies are used in routine clinical practice. Furthermore, in contrast to most published studies [9, 19, 21] that almost exclusively evaluate intermediate lesions in stable patients, our study included a modest percentage (19.5%) of lesions ≥70%. The lesions included in this range of stenoses corresponded mainly to patients referred to the hemodynamics laboratory without prior ischemia evaluation and patients with multivessel disease amenable to surgical revascularization. Also, the RECOPA Study included a nonnegligible proportion of lesions in the acute coronary syndrome scenario (NSTEACS culprit lesion: 15.4%/NSTEACS nonculprit lesion: 10.0%/STEACS nonculprit lesion: 8.4%). These two facts allow us to hypothesize that the range of validation of the technique could extend beyond intermediate lesions in stable patients, which is consistent with the data provided by recent publications [22, 23].

Another strength of our study was the induction of coronary vasodilatation with adenosine—which is the drug most commonly used for this purpose—in contrast to other RFR validation studies involving other vasodilators (nitroglycerin, nicorandil, etc.) [9, 19, 21].

### 4.2. Diagnostic Precision and Agreement between RFR and FFR

The different diagnostic precision measures are related to different aspects of the diagnostic procedures. Correlation between RFR and FFR proved significant (*R*^2^ = 0.81; *p* < 0.001), with values similar to those recently reported by Lee et al. [9] (*R*^2^ = 0.82; *P* < 0.001). The ROC analysis performed in our study showed RFR to have very good discriminating power in measuring ischemia defined as an FFR threshold ≤0.80 (AUC: 0.873 [0.836–0.911]; *P* < 0.001), determining an RFR cut-off point ≤0.88 as being optimal. The results of our ROC analysis are similar to those reported by Svanerud et al. [8] in the pivotal cohort study of RFR versus FFR, showing an AUC of 0.862 (0.834–0.889; *P* < 0.001), though the mentioned authors found the optimal cut-off value to be RFR ≤ 0.89.

However, although the determination of strict cut-off points facilitates clinical decision-making and is supported by the revascularization guides, it may result in simplification of the significance of the different indices. On establishing a comparative dichotomous evaluation of the previously recommended thresholds corresponding to RFR (≤0.89) and FFR (≤0.80), as the main objective of our study, moderate values were obtained for sensitivity (76%), specificity (80%), PPV (68%), and NPV (86%). Thus, the diagnostic accuracy or agreement obtained was only 79%, though this is consistent with the findings of Muroya et al. [19] (81%), who also conducted a dichotomous evaluation of RFR and FFR with the recommended values.

### 4.3. Hybrid RFR-FFR Strategy: Expanding Agreement and Simplifying Physiological Assessment of Coronary Lesions

The above results show that although the correlation between RFR and FFR proved significant and RFR has very high discriminating power, the “all-or-nothing” assessment of RFR levels may result in deficiencies in the diagnostic accuracy of RFR. For this reason, we decided to establish an adenosine administration “grey zone” to increase the predictive values of the technique. This approach, already described for iFR [24], appears to be the most appropriate strategy, for although dichotomous tests are simpler to interpret and are widely used in standard practice, greater precision of the results would be afforded by establishing an intermediate zone in which to assess FFR. Petraco et al. [24] established iFR cut-off points of 0.86 and 0.93, establishing an NPV of 91% for excluding hemodynamic significance of the lesions and a PPV of 92% for identifying functionally significant lesions. In our study, with the established limits of 0.86 and 0.92, similar predictive values would be obtained, with an NPV of 92% and a PPV of 91%. With this hybrid approach, and in accordance with the results of our study, the use of adenosine would be limited to 42% of the physiological studies performed, maintaining agreement superior to 95% with respect to use of FFR alone.

**Figure 5:**
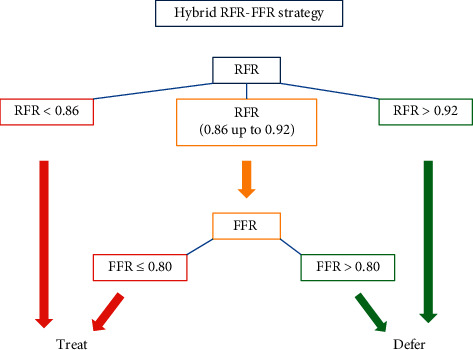
Diagnostic-therapeutic algorithm proposal according to a hybrid RFR-FFR strategy. Initially, the RFR will be performed to assess the hemodynamic significance of the coronary lesions to be evaluated. If the RFR value is inferior to 0.86, the lesion will be treated. In case the RFR value is superior to 0.92, the treatment of the lesion will be deferred. In the intermediate RFR values (from 0.86 to 0.92, with both included), the significance of the coronary lesion will be reclassified by determining the FFR. Lesions with FFR values less than or equal to 0.80 will require treatment. In those lesions with FFR values greater than 0.80, the treatment should be deferred.

The evidence shows the benefits of revascularization guided by the invasive assessment of coronary lesions, and although its use has increased slightly in recent years, it is still little employed in clinical practice—with differences from one country to another [25, 26]. One of the main obstacles to its expanded use in routine practice is the need to administer coronary vasodilators, which is linked to patient discomfort and possible complications [25, 26]. The hybrid RFR-FFR strategy (Figure 5) we recommend shows very good agreement with the reference diagnostic technique and moreover allows an important decrease in drug use. It therefore could facilitate generalization of the invasive assessment of coronary lesions by simplifying the procedures and making them more convenient for the patient and operators.

### 4.4. Limitations

A first limitation of our study is the fact that it is a single-country study, which could limit extrapolation of the findings to other populations. However, its multicenter nature attenuates this limitation by including populations from different geographical areas. Second, the study protocol allowed the induction of maximum hyperemia via both the intravenous and the intracoronary routes, which could influence the study results. However, the use of both forms of hyperemia induction is widely supported in the literature and is considered to be equivalent [27]. Third, the inclusion criteria allowed the enrolment of patients with stable ischemic heart disease or acute coronary syndrome, despite the fact that the invasive assessment of coronary lesions is recommended primarily in patients with stable ischemic heart disease. Nevertheless, these techniques are also used in routine practice in the context of acute coronary syndrome, as supported by the literature [22, 23]. Finally, the percentage of stenoses ≥70% was limited, which reduces validity in reference to angiographically significant lesions. However, the inclusion of lesions ≥70% was greater than that in previous studies [9, 19, 21].

## 5. Conclusions

The agreement between the currently recommended dichotomous values of FRR and FFR is limited. However, the adoption of a hybrid RFR-FFR strategy, with an RFR “grey zone” in which to determine FFR, allows for improved agreement between the two strategies, thus reducing the need for coronary vasodilators.

## Figures and Tables

**Table 1:** Baseline clinical characteristics.

	Patients (*n* = 311)
Age (years), mean (SD)	65.4 (11.5)
Female gender, *n* (%)	61 (19.6%)
BMI (kg/m^2^), mean (SD)	28.2 (4.8)
Hypertension, *n* (%)	220 (70.7%)
Dyslipidemia, *n* (%)	221 (71.1%)
Diabetes mellitus, *n* (%)	
No	200 (64.3%)
Non-insulin-dependent	88 (28.3%)
Insulin-dependent	23 (7.4%)
Smoking, *n* (%)	
Not smoker	126 (40.5%)
Ex-smoker	118 (37.9%)
Current smoker	67 (21.5%)
Prior AMI, *n* (%)	82 (26.4%)
Prior stroke, *n* (%)	24 (7.7%)
Atrial fibrillation, *n* (%)	30 (9.6%)
Peripherical vasculopathy, *n* (%)	31 (10.0%)
COPD, *n* (%)	21 (6.8%)
Chronic kidney disease, *n* (%)	92 (30.6%)
Creatinine (mg/dL), mean (SD)	1.03 (0.61)
Glomerular filtration rate (mL/min/1.73 m^2^), mean (SD)	76.0 (31.1)
Clinical indication, *n* (%)	
Stable ischemic heart disease	206 (66.2%)
NSTEACS culprit lesion	48 (15.4%)
NSTEACS nonculprit lesion	31 (10.0%)
STEACS nonculprit lesion	26 (8.4%)

SD, standard deviation; BMI, body mass index; AMI, acute myocardial infarction; COPD, chronic obstructive pulmonary disease; NSTEACS, non-ST segment elevation acute coronary syndrome; STEACS, ST segment elevation acute coronary syndrome.

**Table 2:** Angiographic characteristics.

	Patients (*n* = 311)
Lesions/patient (*n*), median (minimum-maximum)	1 (1–5)
	Lesions (*n* = 380)
Affected vessel by syntax, *n* (%)	
Left main artery	4 (1.1%)
LAD	225 (59.2%)
LCx	69 (18.2%)
RCA	82 (21.6%)
Percentage of angiographic stenosis (%), mean (SD)	58 (11)
Grouped percentage of angiographic stenosis, *n* (%)	
40–49%	33 (8.7%)
50–59%	121 (31.8%)
60–69%	152 (40.0%)
≥70%	74 (19.5%)
Estimated vessel diameter (mm), mean (SD)	3.02 (0.53)
Length of lesion, *n* (%)	
<12 mm	188 (49.5%)
12–25 mm	148 (38.9%)
>25 mm	44 (11.6%)

SD, standard deviation; LAD, left anterior descending artery; LCx, left circumflex artery; RCA, right coronary artery. Affected segments are shown in Supplementary Material 2.

**Table 3:** Physiological characteristics and final treatment.

	Lesions (*n* = 380)
Adenosine administration, *n* (%)	
Adenosine i.c.	255 (67.1%)
Adenosine e.v.	125 (32.9%)
Guideline catheter size, *n* (%)	
5 French	11 (2.9%)
6 French	368 (96.8%)
7 French	1 (0.3%)
RFR (*n*), median (IQR)	0.91 (0.86–0.95)
RFR results, *n* (%)	
Positive RFR (≤0.89)	152 (40.0%)
Negative RFR (>0.89)	228 (60.0%)
Basal Pd/Pa (*n*), median (IQR)	0.93 (0.90–0.96)
FFR (*n*), median (IQR)	0.84 (0.77–0.89)
FFR results, *n* (%)	
Positive FFR (≤0.80)	136 (35.8%)
Negative FFR (>0.80)	244 (64.2%)
Final treatment by lesions, *n* (%)	
Medical management	256 (67.3%)
PCI-DES	95 (25.0%)
PCI-BMS	4 (1.1%)
PCI-DEB	3 (0.8%)
CABG	22 (5.8%)

RFR, resting full-cycle ratio; IQR, interquartile range; Pd, distal pressure; Pa, aortic pressure; FFR, fractional flow reserve; PCI, percutaneous coronary intervention; DES, drug-eluting stent; BMS, bare metal stent; DEB, drug-eluting balloon; CABG, coronary artery bypass grafting.

## Data Availability

The original database can be made available from the corresponding author upon request.

## Supplementary Materials

Supplementary Materials Supplementary Material 1: general recommendations for FFR and RFR measurements (modified from Achenbach et al. [28] and Svanerud et al. [8]). FFR, fractional flow reserve; RFR, resting full-cycle ratio; Pd, distal pressure; Pa, aortic pressure. Supplementary Material 2: segments affected according to syntax classification. RCA, right coronary artery; LAD, left anterior descending artery; LCx, left circumflex artery. Supplementary Material 3: sensitivity and specificity analyses for overall cohort and stratified by the route of adenosine administration (RFR (≤0.89) and FFR (≤0.80)). A: overall cohort (380 lesions). B: intracoronary adenosine (255 lesions). C: endovenous adenosine (125 lesions).
