# Supplementary material for: Usefulness of the Hybrid RFR-FFR Approach: Results of a Prospective and Multicenter Analysis of Diagnostic Agreement between RFR and FFR—The RECOPA (REsting Full-Cycle Ratio Comparation versus Fractional Flow Reserve (A Prospective Validation)) Study
Source: J Interv Cardiol. 2021 Mar 31;2021:5522707. doi: 10.1155/2021/5522707 (PMC8026323; doi:10.1155/2021/5522707)
Supplement: Supplementary Materials — Supplementary Material 1: general recommendations for FFR and RFR measurements (modified from Achenbach et al. [28] and Svanerud et al. [8]). FFR, fractional flow reserve; RFR, resting full-cycle ratio; Pd, distal pressure; Pa, aortic pressure. Supplementary Material 2: segments affected according to syntax classification. RCA, right coronary artery; LAD, left anterior descending artery; LCx, left circumflex artery. Supplementary Material 3: sensitivity and specificity analyses for overall cohort and stratified by the route of adenosine administration (RFR (≤0.89) and FFR (≤0.80)). A: overall cohort (380 lesions). B: intracoronary adenosine (255 lesions). C: endovenous adenosine (125 lesions). [file 5522707.f1.zip › 5522707.f1/DEFINITIVE RECOPA SUPLEMENTARY MATERIAL 1.docx]

**Supplementary Material 1: General recommendations for FFR and RFR measurements** [Modified from Achenbach et al. (28) and Svanerud et al. (8)].

| **Catheter selection and positioning**  • Use guiding catheters (at least 5F) without side holes.  • Ascertain coaxial catheter cannulation in the coronary ostium.  • Disengage guiding catheter from ostium for pressure calibration, equalisation and recording of Pd/Pa in case of doubts about the catheter potentially obstructing the ostium. |
| --- |
| **Calibration**  • Before starting RFR and FFR measurements, ensure proper zeroing of the aortic pressure (1/3 vs. 2/3 diameter of the chest)  • Flush the guidewire, lay flat when connecting/calibrating and do not move.  • Before equalisation of pressures, advance the guidewire into the coronary artery until the pressure sensor is positioned at the end of the guide catheter.  • Before equalisation of pressures, flush the guide catheter in order to remove the viscous contrast agent.  • Before equalisation of pressures, remove the introducer and close the haemostatic valve.  • Pressure curves are averaged across three-to-five heartbeats. Therefore, pressure equalisation requires some time, and no artefacts should occur during that time. |
| **Positioning of the guidewire**  • Pressure sensor should be positioned in the main vessel directly downstream the most distal lesion.  • Detecting artefacts: the sensor could interact with the vessel wall, especially in cases of a narrow vessel calibre or severe tortuosity.  • Viscous contrast agent in the coronary tree can affect the gradient Pd/Pa. |
| **Hyperemia**  • Prior to advancement of the guidewire, administer intracoronary nitroglicerine (usually 200mcg) to prevent spasms in epicardial vessels.  • In case of evaluating more than one lesion in the same patient, the operator must wait a minimum time of 5 minutes after the adenosine adminnistration to avoid the interference of coronary vasodilatation on RFR values.  - *Medications for hyperemia (exclusively for FFR; not applicable for RFR):*  • Endovenous:  - Adenosine 140 μg / kg / min  - In case of borderline results, an increased dose of adenosine endovenous is possible. However, endovenous doses> 180 μg / kg / min can reduce coronary perfusion and are therefore not recommended.  • Intracoronary:  - Adenosine (up to 200 mcg of intracoronary adenosine for the right coronary artery and up to 300 mcg of adenosine for the left coronary artery). |

FFR, fractional flow reserve; RFR, resting full-cycle ratio, Pd, distal pressure; Pa, aortic pressure.
