# Supplementary material for: Usefulness of the Hybrid RFR-FFR Approach: Results of a Prospective and Multicenter Analysis of Diagnostic Agreement between RFR and FFR—The RECOPA (REsting Full-Cycle Ratio Comparation versus Fractional Flow Reserve (A Prospective Validation)) Study
Source: J Interv Cardiol. 2021 Mar 31;2021:5522707. doi: 10.1155/2021/5522707 (PMC8026323; doi:10.1155/2021/5522707)
Supplement: Supplementary Materials — Supplementary Material 1: general recommendations for FFR and RFR measurements (modified from Achenbach et al. [28] and Svanerud et al. [8]). FFR, fractional flow reserve; RFR, resting full-cycle ratio; Pd, distal pressure; Pa, aortic pressure. Supplementary Material 2: segments affected according to syntax classification. RCA, right coronary artery; LAD, left anterior descending artery; LCx, left circumflex artery. Supplementary Material 3: sensitivity and specificity analyses for overall cohort and stratified by the route of adenosine administration (RFR (≤0.89) and FFR (≤0.80)). A: overall cohort (380 lesions). B: intracoronary adenosine (255 lesions). C: endovenous adenosine (125 lesions). [file 5522707.f1.zip › 5522707.f1/DEFINITIVE RECOPA SUPLEMENTARY MATERIAL 2.docx]

**Supplementary Material 2: Segments affected according to Syntax classification.**

|  | **Lesions (n=380)** |
| --- | --- |
| **Segment affected by Syntax**, *n (%)* |  |
| *1. RCA proximal* | 24 (6.3%) |
| *2. RCA mid* | 33 (8.7%) |
| *3. RCA distal* | 16 (4.2%) |
| *4. Posterior descending from RCA* | 7 (1.8%) |
| *5. Left main* | 4 (1.1%) |
| *6. LAD proximal* | 64 (16.8%) |
| *7. LAD mid* | 128 (33.7%) |
| *8. LAD distal* | 16 (4.2%) |
| *9. First diagonal* | 14 (3.7%) |
| *10. Second diagonal* | 3 (0.8%) |
| *11. Proximal LCx* | 26 (6.8%) |
| *12. Intermediate* | 1 (0.2%) |
| *12a. Obtuse marginal a* | 19 (5.0%) |
| *12b. Obtuse marginal b* | 10 (2.6%) |
| *13. Distal LCx* | 9 (2.4%) |
| *14. Left posterolateral* | 1 (0.2%) |
| *14b. Left posterolateral b* | 1 (0.2%) |
| *15. Posterior descending from LCx* | 2 (0.5%) |
| *16a. Posterolateral from RCA a* | 2 (0.5%) |

RCA, right coronary artery; LAD, left anterior descending artery; LCx, left circumflex artery
