# Supplementary material for: Usefulness of the Hybrid RFR-FFR Approach: Results of a Prospective and Multicenter Analysis of Diagnostic Agreement between RFR and FFR—The RECOPA (REsting Full-Cycle Ratio Comparation versus Fractional Flow Reserve (A Prospective Validation)) Study
Source: J Interv Cardiol. 2021 Mar 31;2021:5522707. doi: 10.1155/2021/5522707 (PMC8026323; doi:10.1155/2021/5522707)
Supplement: Supplementary Materials — Supplementary Material 1: general recommendations for FFR and RFR measurements (modified from Achenbach et al. [28] and Svanerud et al. [8]). FFR, fractional flow reserve; RFR, resting full-cycle ratio; Pd, distal pressure; Pa, aortic pressure. Supplementary Material 2: segments affected according to syntax classification. RCA, right coronary artery; LAD, left anterior descending artery; LCx, left circumflex artery. Supplementary Material 3: sensitivity and specificity analyses for overall cohort and stratified by the route of adenosine administration (RFR (≤0.89) and FFR (≤0.80)). A: overall cohort (380 lesions). B: intracoronary adenosine (255 lesions). C: endovenous adenosine (125 lesions). [file DEFINITIVE RECOPA SUPLEMENTARY MATERIAL 3 REBUTTAL PDF.pdf › 5522707.f1/DEFINITIVE RECOPA SUPLEMENTARY MATERIAL 3 REBUTTAL PDF.pdf]

Supplementary Material 3: Sensistivity and specificity analyses for overall cohort and stratified by the route of adenosine administration [RFR (≤ 0.89) and FFR (≤ 0.80)].

A: Overall cohort (380 lesions).

```
##               FFR
## RFR            pos (<=0.8) neg (>0.8)
##   pos (<=0.89)         104         48
##   neg (>0.89)           32        196
```

```
##           Outcome +    Outcome -      Total
## Test +          104           48        152
## Test -           32          196        228
## Total           136          244        380
##
## Point estimates and 95 % CIs:
## ---------------------------------------------------------
## Apparent prevalence                    0.40 (0.35, 0.45)
## True prevalence                        0.36 (0.31, 0.41)
## Sensitivity                            0.76 (0.68, 0.83)
## Specificity                            0.80 (0.75, 0.85)
## Positive predictive value              0.68 (0.60, 0.76)
## Negative predictive value              0.86 (0.81, 0.90)
## Positive likelihood ratio              3.89 (2.97, 5.09)
## Negative likelihood ratio              0.29 (0.21, 0.40)
## ---------------------------------------------------------
```

B: Intracoronary adenosine (255 lesions).

```
##               FFR
## RFR            pos (<=0.8) neg (>0.8)
##   pos (<=0.89)          73         28
##   neg (>0.89)           20        134
```

```
##           Outcome +    Outcome -      Total
## Test +           73           28        101
## Test -           20          134        154
## Total            93          162        255
##
## Point estimates and 95 % CIs:
## ---------------------------------------------------------
## Apparent prevalence                    0.40 (0.34, 0.46)
## True prevalence                        0.36 (0.31, 0.43)
## Sensitivity                            0.78 (0.69, 0.86)
## Specificity                            0.83 (0.76, 0.88)
## Positive predictive value              0.72 (0.62, 0.81)
## Negative predictive value              0.87 (0.81, 0.92)
## Positive likelihood ratio              4.54 (3.19, 6.47)
## Negative likelihood ratio              0.26 (0.18, 0.39)
## ---------------------------------------------------------
```

C: Endovenous adenosine (125 lesions).

```
##                FFR
## RFR             pos (<=0.8) neg (>0.8)
##   pos (<=0.89)          31         20
##   neg (>0.89)           12         62
```

```
##              Outcome +    Outcome -      Total
## Test +              31           20         51
## Test -              12           62         74
## Total               43           82        125
##
## Point estimates and 95 % CIs:
## ---------------------------------------------------------
## Apparent prevalence                     0.41 (0.32, 0.50)
## True prevalence                         0.34 (0.26, 0.43)
## Sensitivity                             0.72 (0.56, 0.85)
## Specificity                             0.76 (0.65, 0.84)
## Positive predictive value               0.61 (0.46, 0.74)
## Negative predictive value               0.84 (0.73, 0.91)
## Positive likelihood ratio               2.96 (1.93, 4.52)
## Negative likelihood ratio               0.37 (0.22, 0.61)
## ---------------------------------------------------------
```
